# Using a latent growth curve model for an integrative assessment of the effects of genetic and environmental factors on multiple phenotypes

**DOI:** 10.1186/1753-6561-3-s7-s44

**Published:** 2009-12-15

**Authors:** Jemila S Hamid, Nicole M Roslin, Andrew D Paterson, Joseph Beyene

**Affiliations:** 1 Biostatistics Methodology Unit, Child Health Evaluative Sciences, The Hospital for Sick Children Research Institute, 555 University Avenue Toronto, Ontario M5G 1X8, Canada; 2 Program in Genetics and Genomic Biology, The Hospital for Sick Children Research Institute, 555 University Avenue, Toronto, Ontario M5G 1X8, Canada; 3 Dalla Lana School of Public Health, University of Toronto, 155 College Street, Toronto, Ontario M5T 3M7, Canada

## Abstract

We propose the use of latent growth curve model to assess the influence of genetic, environmental, demographic, and lifestyle factors on multiple phenotypes related to coronary heart disease. We model four quantitative traits (systolic blood pressure, high-density lipoprotein, low-density lipoprotein, and triglycerides) simultaneously in a multivariate framework that allows us to study their change over time, assess individual variation, and investigate cross-phenotype relationships. Environmental, demographic, and lifestyle covariates are included at different levels of the model as time-varying or time-invariant, as appropriate. To investigate the change over time attributed to genetic factors, we use candidate markers that have previously been shown to be associated with the quantitative traits. We illustrate our approach using independent observations from the offspring cohort of the Framingham Heart Study data.

## Background

Numerous studies have identified environmental, demographic, and genetic factors that increase the risk of coronary heart disease (CHD). A notable major study that led to the identification of several risk factors for heart disease is the Framingham Heart Study (FHS), which began in 1948. The study provides measurements of major risk factors such as blood pressure and lipid levels taken over a long period of time, offering the opportunity to model developmental trajectories. Very recently, FHS genotyped individuals, which permits researchers to perform genome-wide association and/or linkage analyses to identify potential genetic factors that may influence the development of CHD.

Environmental and genetic variables influencing auantitative traits related to CHD such as systolic blood pressure have been studied extensively. Methods ranging from simple regression to more complicated multilevel models have been used to model the longitudinal aspects of blood pressure and other quantitative traits of interest [1]. However, few studies looked at more than one phenotype simultaneously, and cross-phenotype relationships are not often investigated. In this paper, we consider longitudinal measurements taken from four different phenotypes known to be associated with CHD, namely: systolic blood pressure (SBP), low-density lipoprotein (LDL), high-density lipoprotein (HDL), and triglycerides (TG). We propose the use of latent growth curve (LGC) to simultaneously model these quantitative traits in a multivariate framework that allows us to investigate cross-phenotype correlations as well as to study the effect of environmental, genetic, and other covariates on the change of these phenotypes over time.

## Methods

### Data description

We included data from the FHS offspring cohort provided by Genetic Analysis Workshop 16 (GAW16). We restricted our analysis to independent members of the offspring cohort, which were selected as follows. Starting with the original 1538 families, the Generation 3 cohort was removed, which split the pedigrees into 3379 independent sub-pedigrees. The maximal set of independent samples was obtained, among the samples that belonged to the offspring cohort, consented to have their phenotype data used, and had genotype data, which resulted in 1488 individuals. An additional 171 samples without family data were added for a total of 1659 independent (kinship coefficient = 0) individuals. Among them, 221 individuals had one or more element of missing genotype information and were excluded. We considered time-varying covariates: smoking, hypertension, and cholesterol treatments. Other variables related to CHD including age, sex, body mass index (BMI), and diabetes status were included in the analysis as time-invariant covariates. Selected markers that have been previously identified to be linked and/or associated with the traits are included in the model to account for genetic contribution. The authors have adhered to the data use agreement for FHS data and this agreement has been reviewed and approved by the Research Ethics Board at the Research Institute, The Hospital for Sick Children, Toronto, Canada.

### Marker selection

For the lipid traits, eight markers were selected from genes or gene regions that showed evidence for association with lipoprotein or lipid concentrations and were confirmed in a meta-analysis [2]. Two of these eight markers were not present in either the 500 k or 50 k marker sets, and were also not in strong linkage disequilibrium with any marker. Marker rs11591147 (chromosome 1, in *PCSK9*) was replaced with rs11206510. Marker rs4420638 (chromosome 19, in the *APOE-C1-C4-C2 *gene cluster), which showed a weaker association in Willer et al. [3], was replaced with rs10402771. Similarly, rs1800775 was replaced with rs1150802. No genome-wide study has shown evidence of significant association with either blood pressure or hypertension. However, we include two markers with the smallest *p*-values from a genotypic test in the Wellcome Trust Case-Control Study [4]. Information about the markers is provided in Table 1.

**Table 1:** Selected markers known to be associated with cardiovascular-related traits

Marker	Chromosome	Position (bp)	Nearest gene	Associated trait
rs11206510	1	55,268,627	*PCSK9*	LDL
rs2820037	1	237,503,165	*CHRM3*	SBP
rs693	2	21,085,700	*APOB*	LDL, TG
rs328	8	19,864,004	*LPL*	HDL, TG
rs3890182	9	106,687,476	*ABCA1*	HDL
rs28927680	11	16,124,283	*APOA1 *cluster	HDL, TG
rs1800588	15	56,510,967	*LIPC*	HDL
rs2398162	15	94,631,554	*NR2F2*	SBP
rs1150802	16	55,552,737	*CETP*	HDL
rs10402271	19	50,021,054	*APOE *cluster	LDL

### LGC model

LGC modelling is used to study the effect of genetic and environmental factors on the change of SBP, HDL, LDL, and TG over time. One of the strengths of LGC modelling is that it allows us to study multiple outcomes over time in a multivariate framework, which is particularly useful in investigating the change in the levels of phenotypes simultaneously and assessing cross-phenotype relationships.

Suppose *y*_*pit *_is a measurement taken from individual *i *in pedigree *p *at exam *t*, where *i *= 1, 2, ..., *n*_*p*_, *p *= 1, 2, ..., *k*, *t *= 1, 2, ..., *q*, then the general growth curve model is described as,

where *α*_*pi *_and *β*_*pi *_are the intercept and the slope [5]. Time-varying covariates such as *v*_*pit *_are included in the model at individual level as in Eq. (1), whereas time-invariant covariates such as *w*_*pi *_enter the model through the growth parameters (intercept and slope) as in Eq. (2). Covariates affecting the phenotypes at the pedigree level such as *z*_*p *_are included at the family (or pedigree) level as in Eq. (3). In our case, the measurements corresponding to *y *are SBP, HDL, LDL, and TG, and these four phenotypes are modelled simultaneously as parallel processes. Moreover, we do not have pedigree level parameters *α*_*p *_and *β*_*p *_because we considered unrelated individuals. We analyzed data using Mplus statistical software [6].

## Results

**Figure 1:**
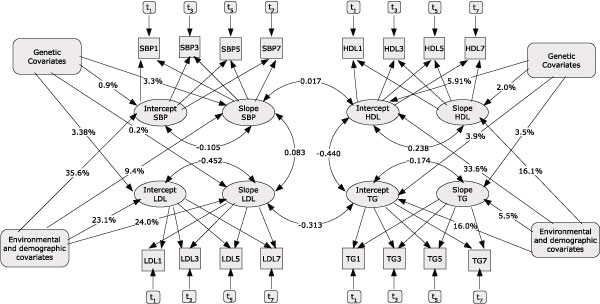
**Path diagram describing growth curve modeling of longitudinal measurements of SBP, HDL, LDL, and TG taken at exams 1, 3, 5, and 7**. The environmental and demographic covariates given on both sides of the path diagram represent the time invariant covariates sex, age, baseline BMI, and diabetes status. Genetic covariates represent the ten selected markers. The numbers on the lines connecting these covariates with the intercepts and slopes are percentages of explained variation and correlations. Paths with one arrow indicate causal relationships whereas those with two show correlations. The boxes contain *t*_*n *_values representing time-varying covariates hypertensive and cholesterol treatments as well as number of cigarettes smoked.

The path diagram given in Figure 1 describes the growth curve used in modeling the longitudinal measurements of SBP, HDL, LDL, and TG. Paths with one arrow represent casual relationships, whereas those with two arrows indicate correlations between the traits involved. For simplicity, we have not included all cross-trait relationships in the diagram; however, the results are provided in Tables 2 and 3. Considerable amount of variation in the intercepts are explained by the time-invariant variables sex, age, baseline BMI, and diabetes status (Table 2). For SBP and HDL, 35.6% and 33.6% of the variations in the intercepts, respectively, are explained by these covariates (Table 2). However, a significant amount of the variations (64.5%, *p*-value < 0.0001 and 66.4%, *p*-value <0.0001, for SBP and HDL, respectively) have not been accounted for. On the other hand, only a small amount of the variation in the slopes is explained by the time invariant covariates, where the largest explained variance is for LDL slopes (24.0%).

**Table 2:** Estimated variance for the latent variables and percentage of variation explained by the time-invariant covariates, genetic covariates, and the combined model

			% Variance explained by		
			
	Mean	Estimated variance	Environmental factors	Genetic factors	Combined model
HDL					
Intercept	52.020	136.926	33.6	3.9	37.5
Slope	0.284	7.860	16.1	3.5	19.6
LDL					
Intercept	125.238	926.508	23.1	3.3	26.7
Slope	1.473	58.518	24.0	0.2	24.0
TG					
Intercept	71.989	3258.821	16.0	18.0	2.1
Slope	22.294	643.769	5.5	7.9	2.0
SBP					
Intercept	119.696	130.198	35.6	0.009	36.3
Slope	2.601	18.524	9.4	0.028	11.8

**Table 3:** Correlations explained by environmental and genetic covariates.^a^

	HDL		LDL		TRG		SBP	
				
	Intercept	Slope	Intercept	Slope	Intercept	Slope	Intercept	Slope
HDL								
Intercept	1.000	0.094	<0.0001	0.003	<0.0001	0.021	0.018	0.001
Slope	0.142	1.000	0.483	<0.0001	0.549	<0.0001	<0.0001	0.058
LDL								
Intercept	**-0.245^b^**	0.040	1.000	<0.0001	<0.0001	0.221	0.961	0.253
Slope	**0.159**	**0.527**	**-0.285**	1.000	0.001	0.001	0.825	0.017
TRG								
Intercept	**-0.318**	-0.031	**0.176**	**-0.179**	1.000	<0.0001	<0.0001	0.139
Slope	-0.094	**-0.608**	0.049	**0.204**	**-0.225**	1.000	0.569	0.008
SBP								
Intercept	0.101	**-0.258**	0.002	-0.012	**0.156**	0.024	1.000	0.020
Slope	**-0.163**	0.138	0.055	0.158	-0.067	**0.134**	-0.163	1.000

^a^Values above diagonal are the corresponding *p*-values.

^b^Bold font indicates significance at *α *= 0.005.

For the genetic factors, the results from our analysis are in agreement with previous association findings indicated in Table 1. We found strong associations between HDL and markers rs28927680 (*p*-value < 0.0001), rs1800588 (*p*-value = 0.002), and rs1150802 (*p*-value < 0.0001) (through the slope). A weak association between HDL slope and marker rs328 was also observed. Markers rs693 and rs10402271 are shown to be strongly associated with the intercept of LDL (both with *p*-value < 0.0001), whereas marker rs11206510 showed a weak association (*p*-value = 0.026). Markers rs28927680 and rs328 are also shown to be strongly associated with the intercept and slope of TG, respectively. For blood pressure, no marker was associated with the intercept of the model; however, a strong association between marker rs1800588 and SBP slope was found (*p*-value = 0.001). This marker is previously liked to HDL [2], but there has not been any study that linked the marker with SBP. It is important to note that markers rs2820037 and rs2398162, with smallest *p*-values from a genotypic test (for SBP) in the Wellcome Trust Case-Control Study [4], did not show any association in our data.

In general, a small amount of variation for all the quantitative traits is attributed to the genetic covariates, where the largest explained variation (3.9%) was for the intercept of HDL (Table 2). The variation in the latent variables explained by the combined model with both the environmental and genetic factors is shown in Table 2. It can be seen that 37.1% of the variation in the slope of HDL is explained by the model; however, a significant amount (86 out of the total 136.93, *p*-value < 0.0001) is left unexplained. Further analysis with more environmental and genetic factors is needed to explain this variation. Moreover, the slope and/or intercept of one or more of the phenotypes could be included as a covariate in the analysis to account for a possible casual dependence between the phenotypes. We plan to consider these analyses in future studies. Here we only investigated the cross-phenotype relationships via correlations. Model estimated correlations for the latent variables are given in Figure 1 using curved, double-arrow lines. Table 3 shows the correlation (along with *p*-values) explained by the environmental and genetic covariates. The residual correlations (data not shown) show that a significant percent of the correlations are not explained by the model, indicating that there are other common factors affecting these phenotypes simultaneously.

## Discussion

Our results show that a significant amount of the variations in the intercepts of the traits are explained by environmental and demographic factors. Moreover, the results identified markers that have been previously associated with the traits. We also found a novel association between marker rs1800588 and SBP. In general, however, only a small percent of the variations in the traits were attributed to the genetic factors.

In our LGC modelling, we considered unrelated individuals (with kinship coefficient = 0) from the offspring cohort of the FHS data. However, one might be interested to know how the intercepts and slopes vary not only at the individual level but also at the family level. Therefore, it is important to use models that take the correlation among family members into account. This will also allow us to explain some of the residual variances and correlations. One can use two approaches in dealing with this challenge 1) adjust for the dependency when the familial correlation is considered as a nuisance parameter and standard errors and goodness-of-fit statistics are estimated using the sandwich estimator or 2) use a two-level LGC model that allows modelling not only average change in the values of the phenotypes over time but also allows us to assess how the these changes vary between individuals in the same family and between families. We plan to address these issues in subsequent studies.

## List of abbreviations used

BMI: Body mass index; CHD: Coronary heart disease; FHS: Framingham Heart Study; GAW16: Genetic Analysis Workshop 16; HDL: High-density lipoprotein; LDL: Low-density lipoprotein; LGC: Latent growth curve; SBP: Systolic blood pressure; SNP: Single-nucleotide polymorphism; TG: Triglyceride

## Competing interests

The authors declare that they have no competing interests.

## Authors' contributions

JSH contributed to the conception and design of the study, carried out the phenotype modeling, and drafted the manuscript. NMR performed marker selection and helped in drafting the manuscript. ADP participated in drafting the manuscript and helped in the biological interpretation of the results. JB contributed to the conception and design of the study, participated in the phenotype modeling and drafting of the manuscript. All authors read and approved the final manuscript.

## References

[B1] Pinnaduwage D Beyene J Fallah S Genome-wide linkage analysis of systolic blood pressure slope using the Genetic Analysis Workshop 13 data BMC Genetics 2003 4 suppl 1 S86 14975154 10.1186/1471-2156-4-S1-S86 PMC1866526

[B2] Kathiresan S Melander O Guiducci C Surti A Burtt NP Rieder MJ Cooper GM Roos C Voight BF Havulinna AS Wahlstrand B Hedner T Corella D Tai ES Ordovas JM Berglund G Vartiainen E Jousilahti P Hedblad B Taskinen MR Newton-Cheh C Salomaa V Peltonen L Groop L Altshuler DM Orho-Melander M Six new loci associated with blood low-density lipoprotein cholesterol high-density lipoprotein cholesterol, or triglycerides in humans Nat Genet 2008 40 189 197 18193044 10.1038/ng.75 PMC2682493

[B3] Willer CJ Sanna S Jackson AU Scuteri A Bonnycastle LL Clarke R Heath SC Timpson NJ Najjar SS Stringham HM Strait J Duren WL Maschio A Busonero F Mulas A Albai G Swift AJ Morken MA Narisu N Bennett D Parish S Shen H Galan P Meneton P Hercberg S Zelenika D Chen WM Li Y Scott LJ Scheet PA Sundvall J Watanabe RM Nagaraja R Ebrahim S Lawlor DA Ben-Shlomo Y Davey-Smith G Shuldiner AR Collins R Bergman RN Uda M Tuomilehto J Cao A Collins FS Lakatta E Lathrop GM Boehnke M Schlessinger D Mohlke KL Abecasis GR Newly identified loci that influence lipid concentrations and risk of coronary artery disease Nat Genet 2008 40 161 169 10.1038/ng.76 18193043 PMC5206900

[B4] Wellcome Trust Case Control Consortium Genome-wide association study of 14,000 cases of seven common diseases and 3,000 shared controls Nature 2007 447 661 678 17554300 10.1038/nature05911 PMC2719288

[B5] Hox J Stoel RD Everitt BS, Howell D Multilevel and SEM approaches to growth curve modeling Encyclopedia of Statistics in Behavioral Science 2005 New York, Wiley 1296 1305

[B6] Muthén LK Muthén BO Mplus Statistical Analysis with Latent Variables User's Guide 2007 5 Los Angeles, Muthén and Muthén http://www.statmodel.com

